# On-site extraction of benzophenones from swimming pool water using hybrid tapes based on the integration of hydrophilic-lipophilic balance microparticles and an outer magnetic nanometric domain

**DOI:** 10.1007/s00604-024-06586-9

**Published:** 2024-08-06

**Authors:** Ahmed Belhameid, Francisco Antonio Casado-Carmona, Adel Megriche, Ángela Inmaculada López-Lorente, Rafael Lucena, Soledad Cárdenas

**Affiliations:** 1 Affordable and Sustainable Sample Preparation (AS2P) Research Group, Departamento de Química Analítica, Instituto Químico para la Energía y el Medioambiente IQUEMA, Universidad de Córdoba, Campus de Rabanales, Edificio Marie Curie, E-14071 Córdoba, Spain; 2 Laboratory of Applied Mineral Chemistry, Faculty of Sciences of Tunis, University of Tunis El Manar, University, Campus El Manar 1, 2092 Tunis, Tunisia; 3 FI-TRACE Group, Department of Chemistry, Faculty of Science, University of the Balearic Islands, Illes Balears, Carretera de Valldemossa Km 7.5, E-07122 Palma de Mallorca, Spain

**Keywords:** On-site extraction, HPLC-MS, Benzophenones, Hydrophilic-lipophilic balance microparticles, Swimming pool waters, Thin-film microextraction, Portable device

## Abstract

An on-site extraction device is presented consisting of scotch tape modified with concentric domains of micrometric hydrophilic-lipophilic balance (HLB) particles surrounded by a ring of nanometric magnetic ones. On the one hand, HLB microparticles are readily available at the surface of the tape, exposed to interact with the target analytes, being responsible for the extraction capacity of the sorptive phase. On the other hand, the presence of magnetic nanoparticles enables the attachment of the modified tape onto a metallic screw via a magnet, which is then coupled to a wireless drill, enabling the stirring of the microextraction device. Both are simply fixed to the cost-effective, flexible, and versatile support, i.e., scotch tape, owing to their adhesive properties. The microextraction device has been applied to the determination of six benzophenones in swimming pool water samples. The variables that may affect the extraction process have been evaluated. Under the optimum conditions and using liquid chromatography-tandem mass spectrometry as the instrumental technique, the method provided a limit of detection of 0.03 µg L^−1^. The intra-day precision, evaluated at three different concentration levels and expressed as relative standard deviation, was lower than 10%, which also comprises the variability within single-use sorptive tapes. The accuracy, calculated with spiked samples and expressed as relative recovery, ranged from 71 to 138%. The method was applied to the analysis of swimming pool water, revealing the presence of such compounds.

**Graphical Abstract:**

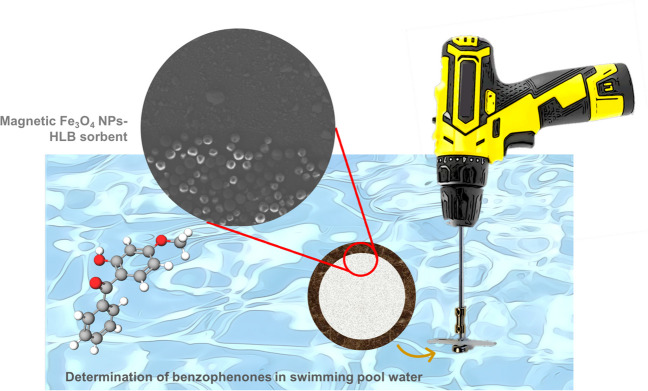

**Supplementary Information:**

The online version contains supplementary material available at 10.1007/s00604-024-06586-9.

## Introduction

The typically low concentration of the target pollutants in environmental compartments makes sample preparation an essential step to guarantee the metrological quality of the results. In this context, sample preparation has been classically developed in the laboratories after the reception and storage of the water samples. However, this conventional strategy limits the development of ambitious sampling campaigns since transporting large sample volumes is challenging from a logistic perspective. Also, the stability of the analytes in the samples during long transportation and storage times compromises the representativeness. On-site extraction has been reported as an efficient alternative to the classical workflow [1, 2], resolving some limitations. The success of this approach relies on the portability of the devices, while the efficiency and affordability of the sorptive phases are vital aspects.

Thanks to their miniaturized size, microextraction techniques have played a role in on-site extraction, and both liquid [3, 4] and sorbent-based microextraction techniques [5, 6] have been reported. Theoretically, sorbent-based microextraction should better stabilize the analytes during transportation/storage, minimizing losses by evaporation, adsorption into vessel walls, or reaction. In reality, despite its importance, the stability of the analytes is not frequently studied, so this statement cannot be unequivocally confirmed. In this sense, solid-phase microextraction (SPME) techniques, which combine solvent-free sampling, extraction, and preconcentration of the analytes into one step, are very powerful for on-site applications. Different SPME approaches have been described in the literature for both equilibrium-based dynamic extraction and diffusion-based passive sampling [5, 7], comprising the use of different configurations, e.g., SPME fibers [8], in-syringe configurations [9, 10], purge and trap [11], or tip microextraction [12], among others. Among the different SPME approaches, thin-film microextraction (TFME), which involves the use of flat substrates in order to enhance the mass transference towards the thin coating, is very suitable for on-site environmental applications [13].

Several substrates, modified with the proper sorbent material by means of, e.g., dip-coating [14], spray, or spin coating [15], have been reported for TFME, which include thin sheets of polymers [16], paper [17–20], fabrics [21], carbon mesh [22], and aluminum foil [23, 24], among others [25]. Within them, stainless steel blades [26] or bolts [27], as well as stir borosilicate disks [28], commercial nylon filtration membranes [1], and modified paper [29], have been applied on TFME-based on-site extraction devices. In addition, hybrid hydrophilic-lipophilic balance (HLB)/polydimethylsiloxane (PDMS) membranes have been incorporated in a drone-based sampler [30]. Herein, we propose the use of scotch tape as a light, cost-effective, and easily modifiable support, enabling the easy attachment of the desired sorbent material.

While in conventional microextraction techniques agitation of the sample usually leads to improved analyte extraction rates, this is not possible for on-site extraction, while stirring or rotation of the sorbent material ensures the efficiency of the extraction procedure [31]. The use of rotated PDMS fibers and thin films was described for the sampling of polycyclic aromatic hydrocarbons (PAHs) in aqueous samples by attaching them to a bench drill [32, 33]. Other portable extraction devices comprise the use of battery-controlled micromotors [34] or small electric motors integrated into glass bottle caps [35, 36]. In previous works, TFME membranes have been fixed to the stirring device composed of a wireless drill coupled to a magnet by means of an iron washer [1], which resulted in a limited mass transference and extraction kinetics. This bottleneck has been overcome by directly fixing the sorbent material to the magnet, thus reducing the diffusion boundary layer, e.g., by using a piece of paper covered by a nanocomposite of silica-coated magnetic nanoparticles (SiO_2_@MNPs) and nylon-6, combining the extraction potential of the polyamide and the magnetic properties of the magnetic nanoparticles (MNPs) [29]. In addition, HLB sorbent microparticles have also been incorporated into a self-adhesive ferrous tape [35], which can be directly attached to the magnet.

In this article, a simple and portable stirring device that allows the integration of sampling and extraction is proposed, which comprises the use of hybrid TFME magnetic tapes integrating concentric micrometric and nanometric domains. The support of the sorbent phase consists of scotch tape, which reduces the cost as compared to commercial ferrous one, while the magnetic character is provided by the attachment of MNPs at the outer nanometric domain, surrounding the HLB active sorbent microparticles, which are located within the inner region, both being incorporated due to adhesive character of the tape. With this approach, the HLB microparticles, which are ideally suited for environmental applications, are completely exposed to the aqueous sample, thus increasing the active surface. The device has been applied to the on-site extraction of several benzophenones in swimming pool water samples.

## Experimental section

### Chemicals and samples

Reagents were provided by Sigma-Aldrich (Madrid, Spain; www.sigmaaldrich.com) unless otherwise specified. All of them were of analytical grade or better. Stock solutions of each analyte (Supplementary Table S1, Electronic Supplementary Material (ESM)), i.e., benzophenone-1 (BP-1), benzophenone-2 (BP-2), benzophenone-3 (BP-3), benzophenone-6 (BP-6), benzophenone-8 (BP-8), and 4-hydroxybenzophenone (4-OH-BP) and the internal standard (oxybenzone-phenyl-d_5_), were prepared in methanol at a concentration of 1 g L^−1^ and stored at 4°C in the dark. Working solutions were prepared daily by dilution in Milli-Q water (Millipore Corp., Madrid, Spain; www.merckmillipore.com) or methanol as needed.

For the preparation of the hybrid sorptive phases, hydrophilic-lipophilic balance microparticles (Oasis HLB, 60 µm particle size, 80 Å pore size) were used as extractant phases. SiO_2_@MNPs were synthesized following the procedure described in the Electronic Supplementary Material using ferrous chloride, ferric chloride, ammonia, and tetraethyl orthosilicate reagents. Transparent adhesive tape (Scotch 550, 19 mm × 33 m, 3M France; https://scotchbrand.3m.com.es/3M/es_ES/scotch-eu/) was used to prepare the sorptive tapes. Swimming pool water samples were obtained from different private pools located at Córdoba (Spain).

### Preparation of the hybrid magnetic sorptive *tapes*

**Fig. 1:**
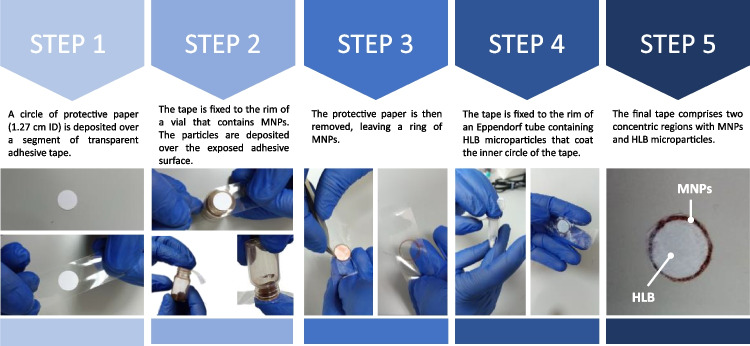
Scheme of the procedure of preparation of the hybrid magnetic sorptive tapes comprising two concentric domains with SiO_2_@MNPs and HLB microparticles

The preparation of the hybrid magnetic tapes is schematically depicted in Fig. 1. Initially, a circle of protective paper (1.27 cm in diameter) is deposited over a segment of transparent adhesive tape (step 1). Then, the tape is fixed to the rim of a vial, with a diameter higher than that of the protective paper, containing SiO_2_@MNPs which are attached over the exposed adhesive surface upon turning the vial upside down (step 2). The protective paper is then removed, leaving a ring of SiO_2_@MNPs (step 3). Finally, the tape is fixed to the rim of an Eppendorf tube, with a diameter of 1.27 cm equal to that of the area previously protected, containing HLB microparticles that coat the inner circle of the tape (step 4). The final tape comprises two concentric regions, whose diameter depends on that of the vial and Eppendorf used to attach the material. The external one contains SiO_2_@MNPs (providing magnetic properties), while the inner one contains HLB (providing sorption capacity) (step 5).

The prepared hybrid magnetic sorptive tapes were characterized by scanning electron microscopy (SEM) by using a JEOL JSM 7800F microscope (www.jeol.com), available at the Central Service for Research Support of the University of Córdoba, which also enables energy-dispersive X-ray (EDX) analysis. Transmission electron microscopy (TEM) images of the magnetic nanoparticles were obtained with a JEOL JEM-1400 microscope, by depositing a drop of the suspension on a copper TEM grid with Carboward forward. Raman spectroscopy measurements were performed with an alpha500 confocal spectrometer (WITec GmbH, Ulm, Germany), by using a 532 nm Nd:YAG laser (green frequency doubled), by using a laser power of 3.68 mW and an integration time of 0.5 s, and accumulating a total of 50 spectra. A 20×/0.4 Zeiss objective was employed. Taking into account the cost-effectiveness of the materials used, new modified tapes were prepared and used for each extraction, which prevented cross-contamination between samples.

### On-site extraction device and procedure

**Fig. 2:**
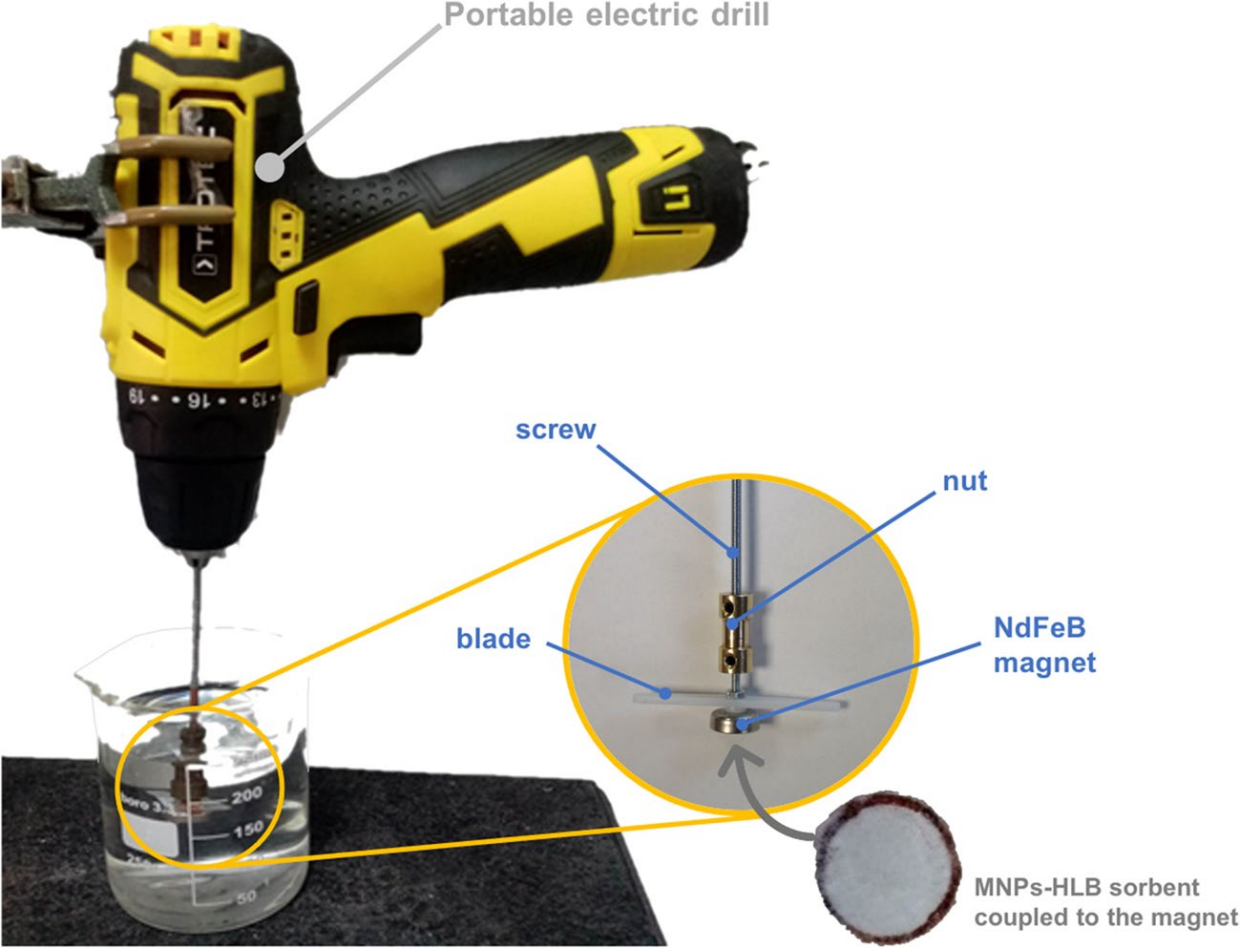
Scheme of the extraction device consisting of a portable electric drill with a screw connected to a NdFeB countersunk pot magnet using a nut. The incorporation of a blade improves the agitation of the unit. The sorptive phase comprising the HLB sorbent and the surrounding SiO_2_@MNPs is attached to the magnet thanks to the magnetic properties of the nanoparticles

The on-site extraction device used in this work is shown in Fig. 2. Details on the components of the device can be found in the Electronic Supplementary Material. Extraction is performed by immersing the extraction device for a certain time in an appropriate volume of sample to which internal standard (i.e., oxybenzone-phenyl-d_5_) is added. The sorptive phases are then stored, and for subsequent analysis, analytes are eluted in methanol. The details on the extraction conditions are provided in the ESM file.

### Liquid chromatographic determination of the target compounds

In this article, two chromatographic methods were employed (described in detail in the Electronic Supplementary Material). A liquid chromatography-UV detection method was used to optimize the extraction procedure, while liquid chromatography-tandem mass spectrometry (LC-MS/MS) was used for the validation. This approach allowed us to minimize the resources by applying the more sophisticated technique only when the extraction procedure was completely understood. The enrichment factor, unaffected by the instrumental technique used, was employed as the optimization parameter.

## Results and discussion

### Characterization of the hybrid magnetic sorptive *tapes*

**Fig. 3:**
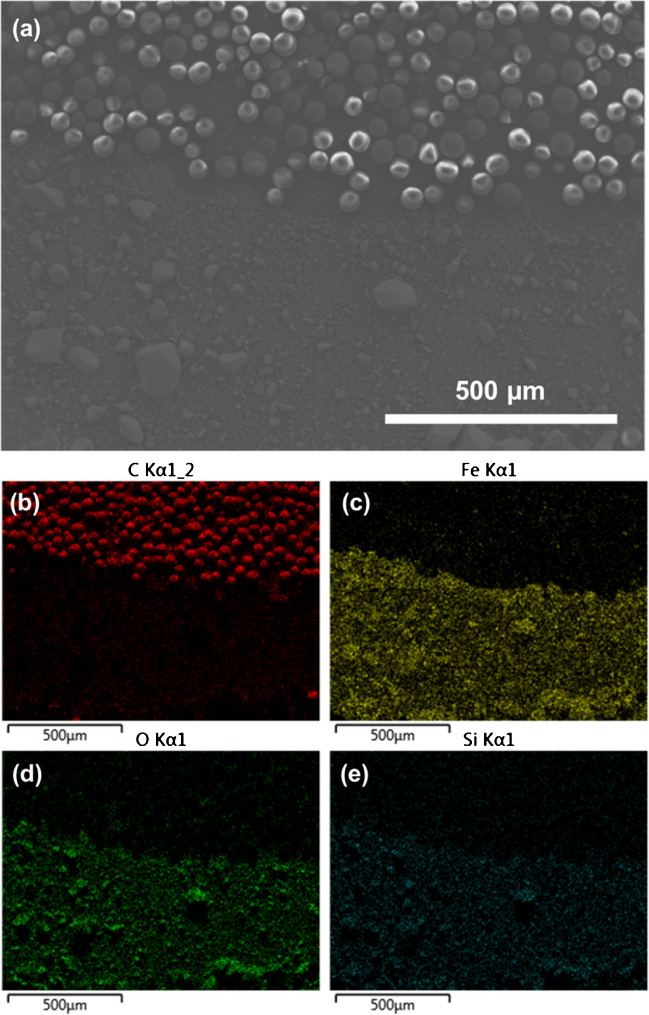
**a** Scanning electron microscopy image at the boundaries between the nanometric outer magnetic domain and the HLB microparticles attached at the inner of the tape. EDX analysis corresponding to the region of the tape shown in the image in which the presence of **b** C, **c** Fe, **d** O, and **e** Si are highlighted in color

The hybrid magnetic tapes prepared, consisting of a scotch tape decorated with SiO_2_@MNPs and HLB microparticles, were characterized by scanning electron microscopy. Figure 3 shows a SEM micrograph of the tape at the boundaries between the nanometric outer magnetic domain and the HLB microparticles attached at the inner of the tape. The average particle size of HLB is about 58 µm in agreement with the value specified by the supplier. The procedure used to prepare the SiO_2_@MNPs yields spherical nanoparticles with diameters of about 11 nm, which tend to form some agglomerates [37], as confirmed by TEM (Supplementary Fig. S1).

Energy-dispersive X-ray analysis of the tape confirmed the presence of the HLB microparticles in the inner region, while the outer region of the circular tape was effectively modified with SiO_2_@MNPs, which are homogeneously distributed within the surface of the scotch tape, as reflected by the presence of Fe, O, and Si in such region. As reported in the literature, magnetite depicts weak chemical stability at pH lower than 4 [38], being stable between 6 and 8.5 [39]. The incorporation of the silica shell covering the MNPs minimizes the oxidation of the nanoparticles at low pH values, thus also preventing the loss of magnetism. The images shown in Fig. 3b–e corroborate the abovementioned distribution of the materials.

In addition, HLB microparticles were also characterized by Raman spectroscopy, the spectra depicting characteristic bands of both pyrrolidone and benzene moieties, as described in more detail in the Electronic Supplementary Material (Supplementary Fig. S2 and S3).

### Optimization of variables affecting the extraction procedure

In this work, different variables that affect the extraction of analytes from aqueous solutions were evaluated univariately, namely, ionic strength, temperature, stirring time, and elution volume. The initial values of each variable for the optimization study were naturally present ionic strength (i.e., 1.43 µS cm^−1^), 25 °C, 15 min, and 1 mL, respectively. For on-site applications, pH, ionic strength, and temperature are relevant parameters, as they may differ from different environmental aqueous compartments. HLB polymeric sorbent is composed of the two monomers, *N*-vinylpyrrolidone and divinylbenzene, providing it with both hydrophilic and lipophilic characteristics, respectively. The presence of the pyrrolidone group renders HLB a suitable sorbent for the extraction of polar compounds. The adsorption of benzophenones on HLB is based on hydrogen bonding interactions with the pyrrolidone group, as well as π-π interactions with the benzene group of the sorbent [40].

The characteristics of the sorbent enabled performing the extraction of the benzophenones at natural sample pH (typically in the range 6–8), without needing to adjust the pH [41, 42], which simplifies the extraction process and would facilitate the possible application of the method directly on the aqueous compartment (e.g., swimming pool, lake, river effluent).

**Fig. 4:**
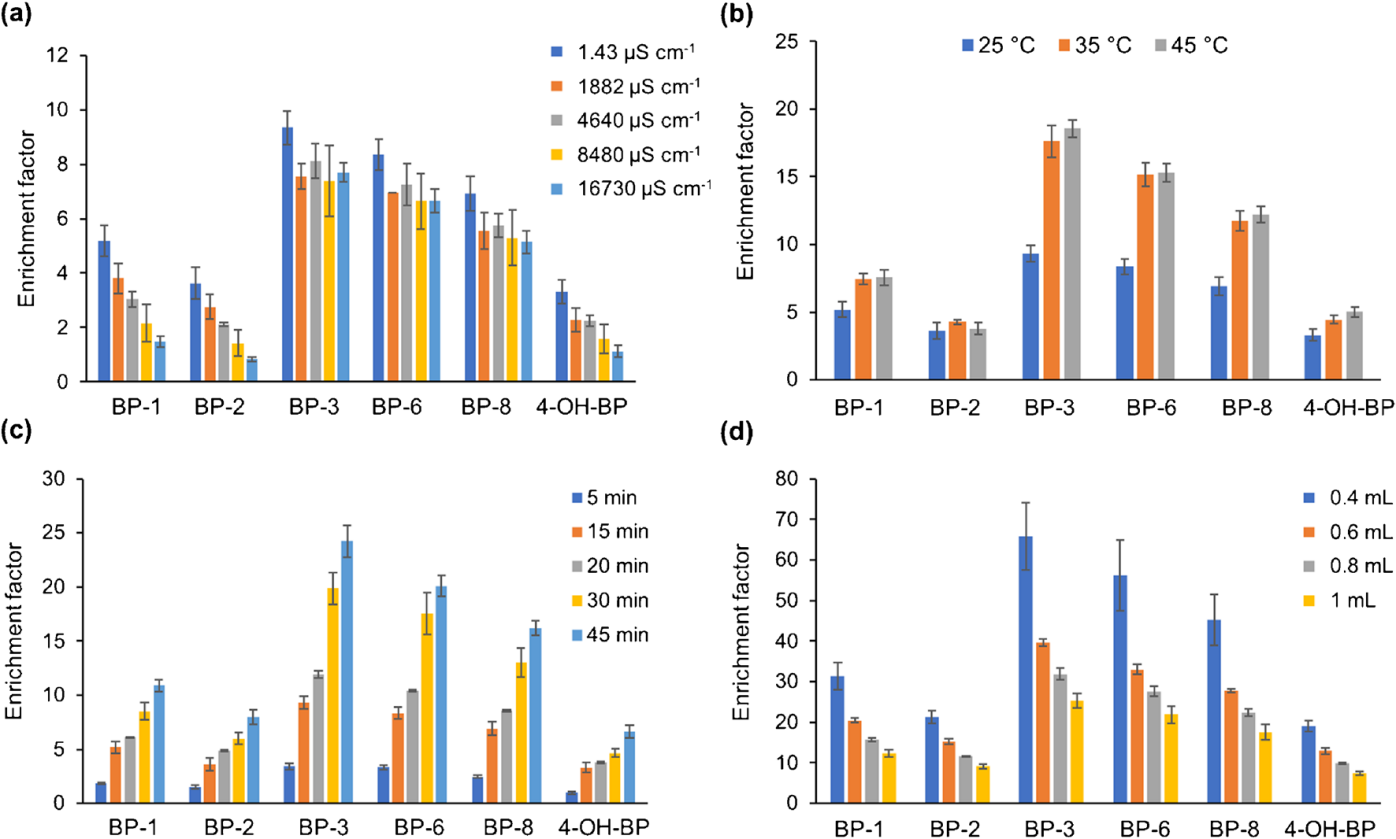
Effect of different variables on the enrichment factor (%) obtained for the target analytes, namely, **a** ionic strength, expressed as conductivity of aqueous solutions containing different amounts of NaCl, **b** temperature, **c** extraction time, and **d** volume of methanol for elution. Initial values of each variable were naturally present ionic strength (i.e., 1.43 µS cm^−1^), 25 °C, 15 min, and 1 mL, respectively

The effect of ionic strength was studied by adjusting the conductivity of the aqueous standards to different levels of conductivity, in the range 1.43–16730 µS cm^−1^, by adding different amounts of NaCl. Generally, the addition of salts may favor the extraction of compounds due to the so-called salting-out effect, reducing the solubility of organic compounds in water, although a high content can also result in an increase in viscosity, which may hinder the diffusion rate. As can be seen in Fig. 4a, in this case, and especially for BP-1, BP-2, and 4-OH-BP, there is a decrease in the extraction yield at high values of salinity. It should be noted that such conductivity levels are above those typically found in river waters, which are in the range 50–1500 µS cm^−1^, for which the diminish in the enrichment factor observed is less pronounced. Although the absolute signal of the analytes is affected by ionic strength, the addition of the internal standard in LC-MS/MS analysis corrects this effect, as also observed in previous studies [35], thus enabling performing the extraction without previous adjustment of conductivity.

As previously mentioned, for on-site extraction, temperature is also important since that of the aqueous compartment will differ along the year and depending on the location. Thus, the effect of this variable was investigated by performing the extraction at three different temperatures, namely, 25 °C, 35 °C, and 45 °C. As depicted in Fig. 4b, the enrichment factor increases with increasing temperature, as it leads to lowering of the viscosity of the solution, thus counteracting the loss of extraction observed by increasing the salinity. Notwithstanding, as previously discussed in the case of ionic strength, the use of an internal standard minimizes and corrects the effect of temperature, making it possible to carry out the extraction procedure without the need of strict control of this parameter.

The extraction performance also depends on other parameters, such as the extraction time, which was evaluated in the range 5 to 45 min, while the sample volume was fixed at 250 mL. Although higher volumes may provide higher sensitivity, we have observed that it can be limited by migration constraints of the analytes from the sample matrix to the sorbent phase. The extraction efficiency increased with time (45 min), providing the best enrichment factor (Fig. 4c). Higher extraction times were not evaluated so as not to excessively lengthen the extraction process and promote a high sample throughput. Finally, the volume of methanol used for the elution of the analytes was studied in the range 0.4 to 1 mL (Fig. 4d), observing, as expected, the higher enrichment with the lower volume.

### Stability of the analytes adsorbed at the hybrid magnetic tape

The developed hybrid magnetic tapes combined with the wireless drill as the agitation source are intended to be used for on-site environmental application, in which the extraction step is carried out at field, while the tapes containing the extracted analytes must be stored and transported until the laboratory, where the subsequent elution and analysis will be performed. Thus, to ensure the representativeness of the results obtained, it is important to evaluate the stability of the analytes on the tape upon storage time. For this purpose, extractions were conducted with 18 independent tapes, which were then dried with a tissue. Three of them were immediately eluted and analyzed while the remaining were stored refrigerated for different periods of time, until 11 days, after which elution and analysis were carried out. Supplementary Fig. S4 depicts the evolution of the peak area obtained for the different analytes over time, observing that the analytes remained stable during this period. One-way analysis of variance (ANOVA) tests have been performed to evaluate whether there are significant differences within the days in which measurements were carried out, observing that there are no significant differences. For example, the calculated values of *F* for BP-1, BP-3, BP-8, and 4-OH-BP were 1.572, 2.436, 2.495, and 1.455, which are below the critical value of *F* (i.e., 3.106) for *α* = 0.05, and 5 and 12 degrees of freedom between groups and within groups, respectively.

### Analytical figures of merit of the method and application to real samples

The analytical figures of merit of the method under the selected conditions were evaluated in terms of linearity, sensitivity (expressed as the limit of detection (LOD) and limit of quantification (LOQ)), precision (expressed as relative standard deviation (RSD)), and accuracy (i.e., relative recovery of blank swimming pool samples spiked with the target analytes at different concentration levels). Table 1 summarizes the values obtained during the validation. Aqueous standard solutions in triplicate at six different concentration levels of the analytes and a fixed concentration of IS of 5 µg L^−1^ were submitted to the analytical process. The calibration curves were obtained by plotting the peak area of the analytes divided by that of the internal standard vs. concentration fitted linearly (*R*^2^ > 0.9984) (Supplementary Fig. S5). The LOD and LOQ were calculated, considering a signal-to-noise ratio of 3 and 10, respectively, obtaining values of 0.03 µg L^−1^ and 0.1 µg L^−1^ for all the analytes. Linearity was maintained between the LOQ and 25 µg L^−1^. The precision was evaluated at three concentration levels, i.e., 0.1 µg L^−1^, 5 µg L^−1^, and 25 µg L^−1^, by carrying out independent replicates (*n* = 5) of the whole analytical procedure, including extraction, elution, and analysis. The RSD values obtained were lower than 10% obtained for BP-2 at the minor concentration level. It should be noted that although the RSD was evaluated intra-day, as the modified tapes were not reused to prevent cross-contamination since the fabrication method is simple, fast, and cost-effective, the variability in the preparation of the different tapes is also considered within the precision. Moreover, the accuracy of the method was also studied at the three concentration levels, by spiking blank swimming pool samples. The relative recoveries found were in the range of 71–138%.

**Table 1:** Analytical figures of merit of the method for the determination of benzophenones

Analyte	LOD (µg L^−1^)	LOQ (µg L^−1^)	*R*^2^	Linear range (µg L^−1^)	RSD intra-day, *n* = 5 (%)			Accuracy (% relative recovery)		
					0.1 µg L^−1^	5 µg L^−1^	25 µg L^−1^	0.1 µg L^−1^	5 µg L^−1^	25 µg L^−1^
Benzophenone-1	0.03	0.1	0.9993	LOQ–25	9.5	7.1	5.0	102 ± 10	85 ± 6	96 ± 5
Benzophenone-2			0.9998		10.0	8.1	3.2	93 ± 9	80 ± 6	86 ± 3
Benzophenone-3			0.9999		8.0	5.0	0.8	95 ± 8	93 ± 5	93 ± 1
Benzophenone-6			0.9998		4.8	2.3	2.6	133 ± 6	117 ± 3	114 ± 3
Benzophenone-8			0.9997		4.8	4.3	1.3	100 ± 5	108 ± 5	111 ± 1
4-Hydroxybenzophenone			0.9984		5.6	9.6	3.9	71 ± 4	127 ± 12	138 ± 5

The proposed method was employed for the analysis of aqueous samples obtained from different private swimming pools in Córdoba, Spain. Benzophenones are UV filters commonly present in many personal care products including sunscreen, thus, easily reaching environmental aqueous compartments, as well as seawater and swimming pool water. These compounds have been classified as endocrine-disrupting chemicals, their presence raising concern due to their possible estrogenic activity. Notwithstanding, the use of some of them as UV filters in cosmetic products is allowed by the Commission Regulation of the European Union, which has recently revised the potential effect of BP-3, thus regulating the concentration levels allowed in different cosmetic products, e.g., for body products, including propellant and pump sprays, and the maximum concentration in ready use preparation has been set as 2.2% [43]. However, cosmetic products containing BP-3 according to the previous regulation (EC), which allows 10% content level of BP-3 [44], have been made available on the Union market until July 2023. Such EU regulation establishes limitations on the content of benzophenone-4 and benzophenone-5, while no information is found about the other compounds of the family. Table 2 shows the results obtained from the analysis of the swimming pool samples. BP-3 and BP-6 are the two more frequently detected in the samples, four samples containing them above the LOQ, while they have been also detected in another sample. In addition, BP-8 and 4-OH-BP were present in three samples. On the other hand, BP-1 was found only in one sample, while BP-2 was not detected in any of them.

**Table 2:** Analysis of real aqueous samples of swimming pool water

Sample	BP-1 (µg L^−1^)	BP-2 (µg L^−1^)	BP-3 (µg L^−1^)	BP-6 (µg L^−1^)	BP-8 (µg L^−1^)	4-OH-BP (µg L^−1^)
Swimming pool 1	–	–	7.1 ± 0.4	1.7 ± 0.1	2.9 ± 0.1	0.66 ± 0.04
Swimming pool 2	–	–	0.14 ± 0.01	0.31 ± 0.01	0.126 ± 0.005	–
Swimming pool 3	0.38 ± 0.02	–	0.33 ± 0.02	0.39 ± 0.02	–	2.4 ± 0.1
Swimming pool 4	–	–	0.87 ± 0.07	0.87 ± 0.04	–	2.6 ± 0.1
Swimming pool 5	–	–	Detected	Detected	0.122 ± 0.005	–
Swimming pool 6	–	–	–	–	–	–
Swimming pool 7	–	–	Detected	Detected	–	–
Swimming pool 8	–	–	Detected	Detected	–	–

Results are expressed as the mean value of concentration ± standard deviation

Finally, the performance of the method was compared with other methods described in the literature for the determination of benzophenones in aqueous samples. As can be seen in Table 3, methods involving exhaustive solid-phase extractions (SPEs), when coupled to chromatography and mass spectrometric analysis, yield very low LODs in the low ng L^−1^ range. Notwithstanding, the sensitivity reached with this method is in a similar range to that achieved with dispersive SPE, in which analyte-sorbent interactions are improved, while it improves that obtained when using an on-site extraction device with nylon membranes. The precision and accuracy levels are comparable to that of the other methods reported. An advantage of the proposed approach is the easiness in the preparation of the sorptive phase, which is based on cost-effective materials, while the consumption of reagents and solvents is limited.

**Table 3:** Comparison of the performance of the proposed method with methods described in literature for the determination of benzophenones in environmental water samples

Analytes (BPs)	Sample	Extraction technique	Sorbent material	Instrumental technique	LOD (ng L^−1^)	RSD (%)	Accuracy (%)	Ref.
BP-1, BP-3, BP-8	Groundwater, river, WWTP effluent	SPE	HLB	GC-MS/MS	0.3–1.0	<11	80–106	[45]
BP-1, BP-2, BP-3, BP-4, BP-6, BP-8	Wastewater, river	SPE	HLB	LC-MS/MS	<1–32	<13.5	83–105	[42]
BP, 4-OH-BP, BP-1, BP-3, BP-8, EtBP, AcBP, iPrBP	Tap, surface water	SPE-MAE	HLB	GC-MS	0.1–1.9	<9.6	98.4–110	[46]
BP-1, BP-8, BP-3	Influent and effluent STP, river	SPE	HLB/Bond Elut Plexa	UHPLC-MS/MS	1.0–2.0	<18	56–97	[47]
BP, BP-1, BP-2, BP-3, BP-4	Surface water, effluent wastewater	SPE	HLB	UHPLC-MS/MS	2.6–10.6 pg	–	70–120	[41]
BP-4, BP-3	River, sea, treated and raw wastewater	SPE	HLB	LC-MS/MS	500–1500	1.1–1.4	71–108	[48]
BP-1, BP-2, BP-3, BP-8, 4-OH-BP	WWTP samples	SPE	HLB	LC-MS/MS	41–67	< 13.8	70–116	[49]
BP-3, BP-4	Wastewater, swimming pool	On-line SPE	C18	LC-UV	2510–4670	<8.7	65–107	[50]
BP-3, BP-1, BP-8, 2-OH-BP, 3-OH-BP, 4-OH-BP	MWTP effluent, river	DµSPE	HLB	GC-MS	0.5–2.0	<11	87–95	[51]
BP-1, BP-2, BP-4, 4-OH-BP, 4DHB	Lake, river	Magnetic DµSPE	Fe_3_O_4_-graphitized carbon black	UHPLC-MS/MS	8–10	<15	75–118	[52]
BP-1, BP-3, 4-OH-BP, BP-6, BP-8	Swimming pool	Magnetic DµSPE	Fe_3_O_4_@GO	LC-MS/MS	2500–8200	<9.8	86–105	[37]
BP-1, BP-2, BP-3, BP-6, BP-8, 4-OH-BP	Sea, river, swimming pool	Magnetic DµSPE	Fe_3_O_4_@SiO_2_@MIM-PF_6_	LC-MS/MS	160–1210	<8.3	87–99	[53]
BP-1, BP-3, BP-6, BP-8, 4-OH-BP	Swimming pool	Stir membrane	Nylon membrane	LC-MS/MS	100	<9.9	74–111	[1]
BP-1, BP-2, BP-3, BP-6, BP-8, 4-OH-BP	Swimming pool	TFME	Magnetic tape modified with HLB	LC-MS/MS	30–100	<10.0	71–138	This work

*MWTP* municipal wastewater treatment plant, *DµSPE* dispersive micro solid-phase extraction, *SPE* solid-phase extraction, *WWTP* wastewater treatment plant, *MAE* microwave-assisted extraction, *EtBP* 3-ethylbenzophenone, *AcBP* 3-acetylbenzophenone, *iPrBP* 3-i-propylbenzophenone, *4DHB* 4,4′-dihydroxybenzophenone, *GO* graphene oxide, *STP* sewage treatment plant

## Conclusions

In this work, an on-site extraction device based on the use of a wireless drill coupled to a metallic screw to which the sorptive phase is attached through a magnet has been developed. The sorbent material consists of a scotch tape decorated with two different domains, i.e., an outer ring of SiO_2_@MNPs, which confers the tape with magnetic characteristics, and the inner core in which HLB microparticles are retained acting as the sorbent material for the extraction of the target analytes. Both SiO_2_@MNPs and HLB microparticles have been physically bonded at the surface of scotch tape by taking advantage of their adhesive character. HLB microparticles are ideally suited for the extraction of many different environmental pollutants, while the subsequent analysis by LC-MS/MS provides the method with selectivity.

The stability of the extracted analytes has been demonstrated, being possible to perform the extraction on-site, while the sorbents can be stored and analyzed later on at the laboratory, which implies an advantage as regards other extraction techniques. This approach enables performing the sampling at different locations within the same environmental compartment easily and transporting just the sorbent. The cost-effectiveness and easy preparation of the sorbent material enables the preparation of an individual sorbent for each extraction, thus preventing cross-contamination. Moreover, the use of an internal standard avoids the strict control of critical variables such as temperature or conductivity of the sample. In this work, swimming pool water samples have been used as representative examples of environmental water, comprising water with different conductivities as both chlorine and saline chlorination pool samples have been analyzed. The on-site extraction scheme developed can be easily applied to other aqueous compartments such as sea, lake, or river water. In addition, given the widespread use of HLB sorbent, the proposed method can be easily extended to other environmental pollutants. The main limitation of the on-site extraction device is that it can be applied to aqueous samples to which the operator has access, while the coupling to remote control devices would enable application also to hard-to-reach locations.

## Supplementary Information

Below is the link to the electronic supplementary material.

Supplementary file1 (DOCX 53622 KB)

## Data Availability

Data will be made available on request.
