# Supplementary material for: On-site extraction of benzophenones from swimming pool water using hybrid tapes based on the integration of hydrophilic-lipophilic balance microparticles and an outer magnetic nanometric domain
Source: Mikrochim Acta. 2024 Aug 6;191(9):513. doi: 10.1007/s00604-024-06586-9 (PMC11303577; doi:10.1007/s00604-024-06586-9)
Supplement: Supplementary file 1 — Supplementary file1 (DOCX 53622 KB) [file 604_2024_6586_MOESM1_ESM.docx]

Electronic Supplementary Material

**On-site extraction of benzophenones from swimming pool water using hybrid tapes based on the integration of hydrophilic-lipophilic balance microparticles and an outer magnetic nanometric domain**

Ahmed Belhameid^a,b^, Francisco Antonio Casado-Carmona^a,c^, Adel Megriche^b^, Ángela Inmaculada López-Lorente^a*^, Rafael Lucena^a*^ and Soledad Cárdenas^a^

^a^Affordable and Sustainable Sample Preparation (AS_2_P) research group, Departamento de Química Analítica, Instituto Químico para la Energía y el Medioambiente IQUEMA, Universidad de Córdoba, Campus de Rabanales, Edificio Marie Curie, E-14071, Córdoba, Spain.

^b^Laboratory of Applied Mineral Chemistry, Faculty of Sciences of Tunis, University of Tunis El Manar, University Campus El Manar 1, 2092 Tunis, Tunisia.

^c^FI-TRACE Group, Department of Chemistry, Faculty of Science, University of the Balearic Islands, Carretera de Valldemossa Km 7.5, E-07122, Palma de Mallorca, Illes Balears, Spain.

Corresponding authors email: [q32loloa@uco.es](mailto:q32loloa@uco.es) (A.I. López-Lorente), [q62luror@uco.es](mailto:q62luror@uco.es) (R. Lucena)

**Experimental section**

**Table S1.** List of the compounds determined in this work, along with the abbreviation, the corresponding structures, n-octanol/water partition ratio or coefficient and pKa value.

| **Benzophenone abbreviation** | **Compound** | **Structure** | **Log K_OW_^a^** | **pKa^b^** |
| --- | --- | --- | --- | --- |
| BP-1 | 2,4-dihydroxybenzophenone | 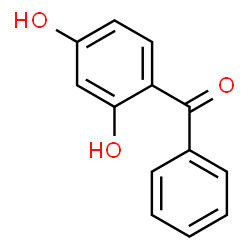 | 3.17 | 7.72 |
| BP-2 | 2,2',4,4'-Tetrahydroxybenzophenone | 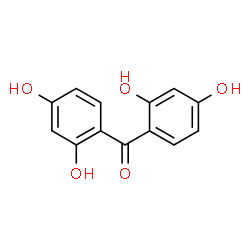 | 3.16 | 6.98 |
| BP-3 | 2-hydroxy-4-methoxybenzophenone or oxybenzone | 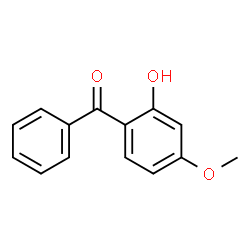 | 3.64 | 7.56 |
| BP-6 | 2,2'-Dihydroxy-4,4'-dimethoxybenzophenone | 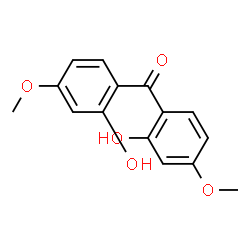 | 4.10 | 6.81 |
| BP-8 | **2,2′-dihydroxy-4-methoxybenzophenone** | 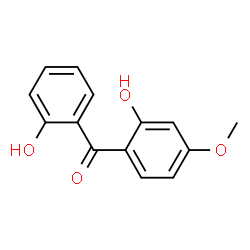 | 3.93 | 7.11 |
| 4OH-BP | 4-hydroxybenzophenone | 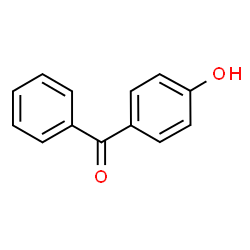 | 2.87 | 8.14 |

^a^Data obtained from ChemSpider (<http://www.chemspider.com/>)

^b^Data obtained from ChemicalBook (<http://www.chemicalbook.com/>).

**Synthesis of the magnetic nanoparticles**

Silica-coated MNPs (SiO_2_@MNPs) were used to provide magnetic behavior to the sorptive tapes. These particles were synthesized following a protocol previously used in our group [1]. Initially, 24 g of FeCl_3_.6H_2_O and 9.8 g FeCl_2_.4H_2_O were dissolved in 100 mL of Milli-Q water under nitrogen flow. The solution was stirred for 30 min at 80°C in a water bath, followed by the dropwise addition of 50 mL of ammonia (25% w/v) forming a black precipitate of magnetic iron oxide nanoparticles (Fe_3_O_4_). The nanoparticles were recovered with a magnet and washed with water to remove unreacted chemicals. The nanoparticles were then dried at 80°C overnight. The dried nanoparticles were coated with a silica shell using tetraethyl orthosilicate (TEOS) by adding 1 g of the nanoparticles to a beaker containing 50 mL of ethanol and 4 mL of Milli-Q water. The pH of the mixture was adjusted to 9.0 using ammonia and subsequently 2 mL of TEOS was added. The resulting dispersion was stirred for 12 h at inert atmosphere. The SiO_2_@MNPs were recovered using a magnet, washed, and dried at 80°C for 12 h.

**On-site extraction device and procedure**

The on-site extraction device employed is based on that described in a previous publication, by using a different sorptive phase [2]. It comprises a wireless drill as the agitation source. A NdFeB magnet (Supermagnete, Gottmadingen, Germany, www.supermagnete.de), which holds the sorptive phase during the extraction, is attached to the drill by a metallic screw. Due to its magnetic character, the sorptive phase can be easily attached to the magnet and remains mechanically stable during the extraction.

For on-site extraction, 250 mL of the sample is taken into a beaker and internal standard (i.e., oxybenzone-phenyl-d_5_) is added to reach a final concentration of 5 µg L^-1^. The extraction device is immersed and stirred for 45 min to isolate the target analytes. After the extraction, the membrane is dried using tissue paper and finally introduced into an Eppendorf where it is stored until its final analysis. Before the analysis, the membranes are eluted with 0.4 mL of methanol under vortex stirring for 10 min.

**Liquid chromatography with UV detection method**

For LC-UV analysis, an HP1100 series liquid chromatograph (Agilent, Palo Alto, CA) equipped with a high-pressure binary pump for mobile phase delivery, an autosampler, and a single-wavelength photometer was employed. HP Chem-Station software was used for performing data analysis. The mobile phase employed for the chromatographic separation consisted of a mixture of (A) an aqueous solution 3% (v/v) of acetic acid and (B) acetonitrile, operating under gradient mode, i.e., from 0 to 15 min, the composition of B increased from 50 % to 60 %. A re-equilibration period of 4 minutes was established after each analysis. Separations were carried out on a LiChrosorb C18 column (4.6 x 250 mm) (Análisis Vinicos, Tomelloso, Spain). The injection volume was 20 µL, the flow rate was kept constant at 1 mL/min and the analytes were measured at 313 nm.

**Liquid chromatography-tandem mass spectrometry method**

LC-MS/MS analyses were carried out on an Agilent1260 Infinity HPLC system (Agilent, Palo Alto, CA, USA, www.agilent.com) using an Eclipse XDB-C18 (4.6 mm x 150 mm, 5 m) column from Agilent. It was preserved using a guard column (0.2 m filter, 2.1 mm). The mobile phase consisted of a mixture of 0.1% ammonium formate aqueous solution and acetonitrile in a 25/75 (v/v) ratio, which flowed at 0.5 mL min^-1^. The injection volume was 5 µL of standards or extracts. Quantification was performed on an Agilent 6420 Triple Quadrupole MS with an electrospray source. The mass spectrometer settings for the detection and quantification of the analytes studied as well as those of the internal standard, which are shown in Table S2, were fixed to improve the multiple reaction monitoring (MRM) signals. The flow rate and the temperature of the drying gas (N_2_) were 9 L/min and 350ºC, respectively. The nebulizer pressure was 30 psi, and the capillary voltage was kept to 3000 V in positive mode.

**Table S2.** Multiple reaction monitoring transitions of the analytes and the internal standard compounds.

| **Compound** | **Precursor ion (m/z)** | **Fragmentor**  **voltage (V)** | **Product ion**  **(m/z)** | **Collision**  **energy (V)** | **Quantitation**  **transition** |
| --- | --- | --- | --- | --- | --- |
| BP-1 | 215 | 115 | 137 | 20 | 215🡪137 |
|  |  |  | 105 | 20 |  |
| BP-2 | 247 | 110 | 137 | 15 | 247 🡪137 |
|  |  |  | 81 | 35 |  |
| BP-3 | 229.1 | 125 | 151 | 20 | 229.1🡪151 |
|  |  |  | 105 | 20 |  |
| BP-6 | 275 | 120 | 151 | 20 | 275🡪151 |
|  |  |  | 95.1 | 45 |  |
| BP-8 | 244.9 | 110 | 151 | 20 | 244.9🡪121 |
|  |  |  | 121 | 20 |  |
| 4OH-BP | 199 | 105 | 121 | 20 | 199🡪121 |
|  |  |  | 77.2 | 45 |  |
| Ozybenzone-(phenyl-d_5_) | 234 | 135 | 150.9 | 20 | 234🡪150.9 |
|  |  |  | 110 | 25 |  |

**Results and discussion**

**Fig. S1** Transmission electron microscopy (TEM) image of the prepared magnetic nanoparticles.


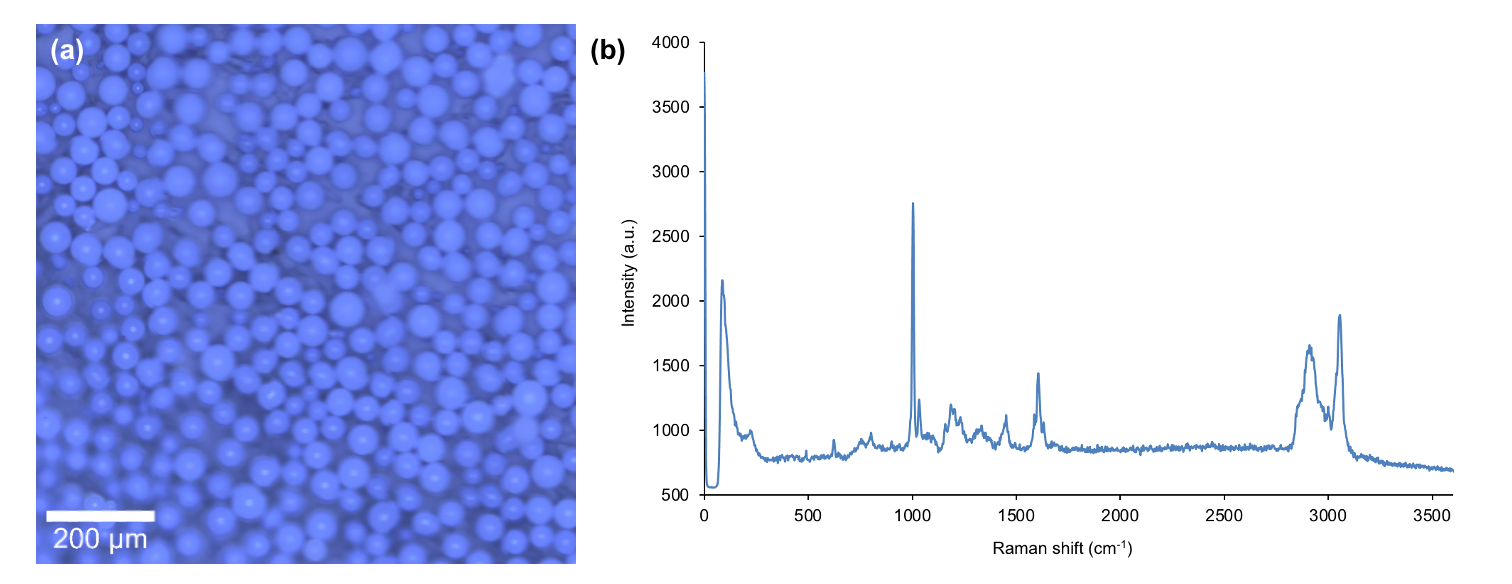


**Fig. S2** (a) Optical microscope photograph, and (b) Raman spectrum of the HLB microparticles attached to the surface of scotch tape.


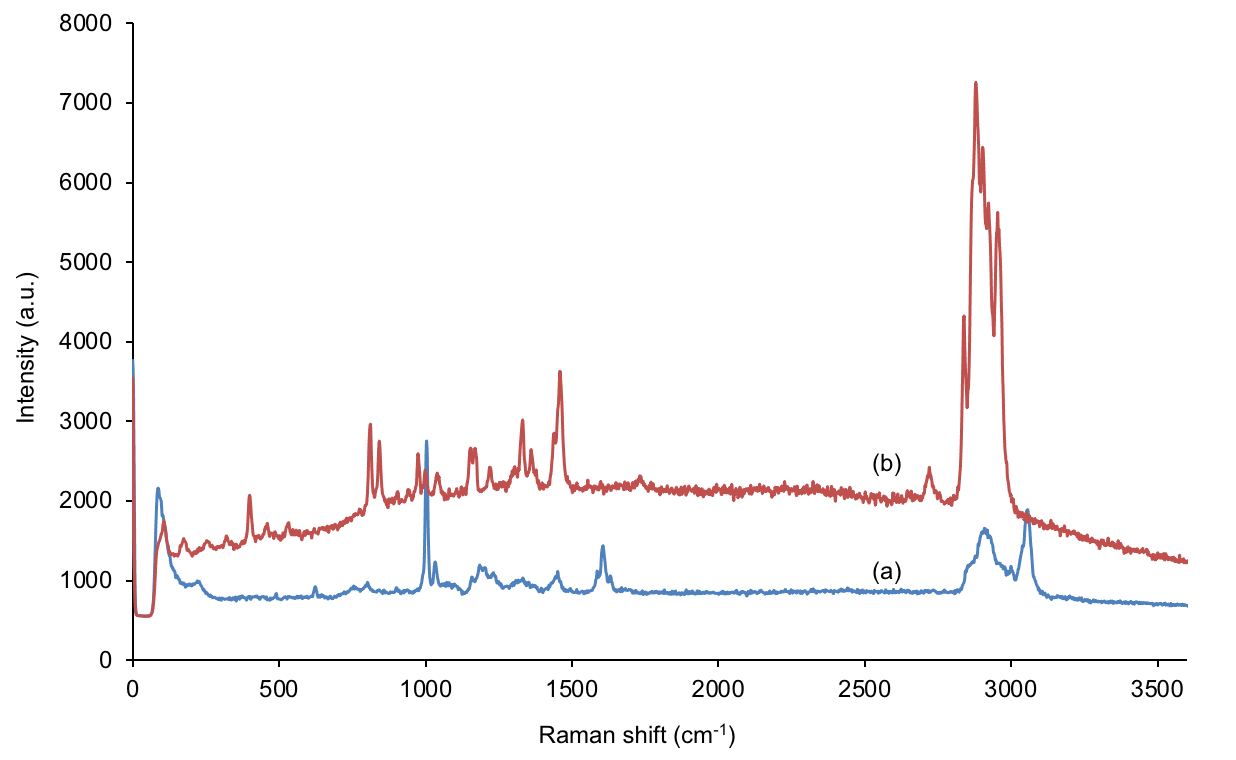


**Fig. S3** Raman spectra of (a) HLB microparticles, (b) scotch tape.

Figure S2a depicts the optical microscope image of the HLB microparticles deposited on the scotch tape, in which a homogeneous distribution of the microparticles within the sorptive phase can be observed. The Raman spectrum of the HLB microparticles (Fig. S2b) contains characteristic features of both the pyrrolidone ring as well as that of the presence of benzene ring within the divinylbenzene monomers. In this sense, the most relevant bands in the spectrum can be ascribed to the vibration of the phenyl ring at 1005 cm-1 [2]. According to the literature, the band at ca. 1608 cm^-1^ can be ascribed to C=O and C-N stretching of vinylpyrrolidone, while the wide band around 1462 cm^-1^ can be due to CH_2_ scissor vibrations [3]. The CCC stretch vibrations of the benzene ring may be the small shoulder at ca. 1586 cm^-1^ [4]. On the other hand, the broad band in the range of 2830-2989 cm^-1^ comprises C-H stretching modes of the pyrrolidone ring, as well as those of the backbone [3] and C-H stretching vibrations of benzene, in this case the C-H symmetrical stretching of benzene leading to the band at ca. 3063 cm^-1^ [4].The Raman spectrum obtained for HLB has been compared to that of the scotch tape used as support (Fig. S3) observing that these main peaks are due to the HLB microparticles, that were focused at the focal plane of the Raman microscope.

**
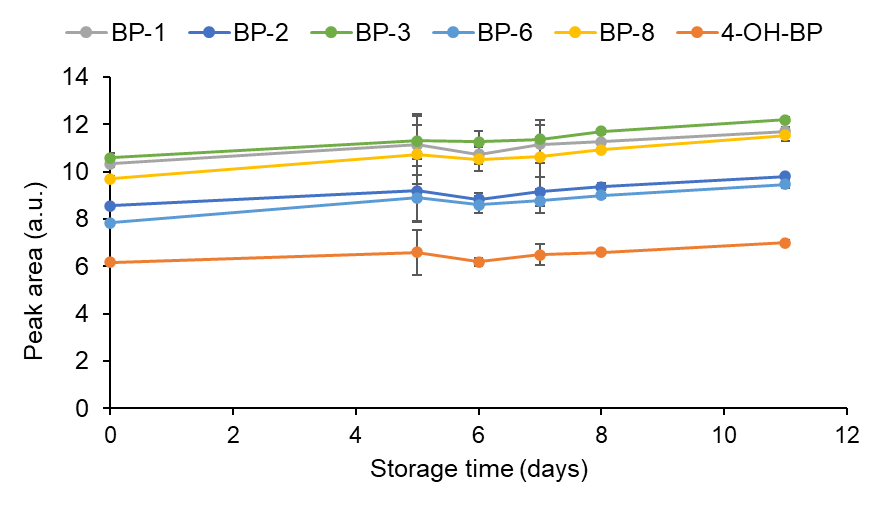
**

**Fig. S4** Study of the stability of the benzophenones once extracted on the hybrid magnetic tape upon storage time (n=3). The tapes were eluted after different storage time and analyzed via LC-UV.


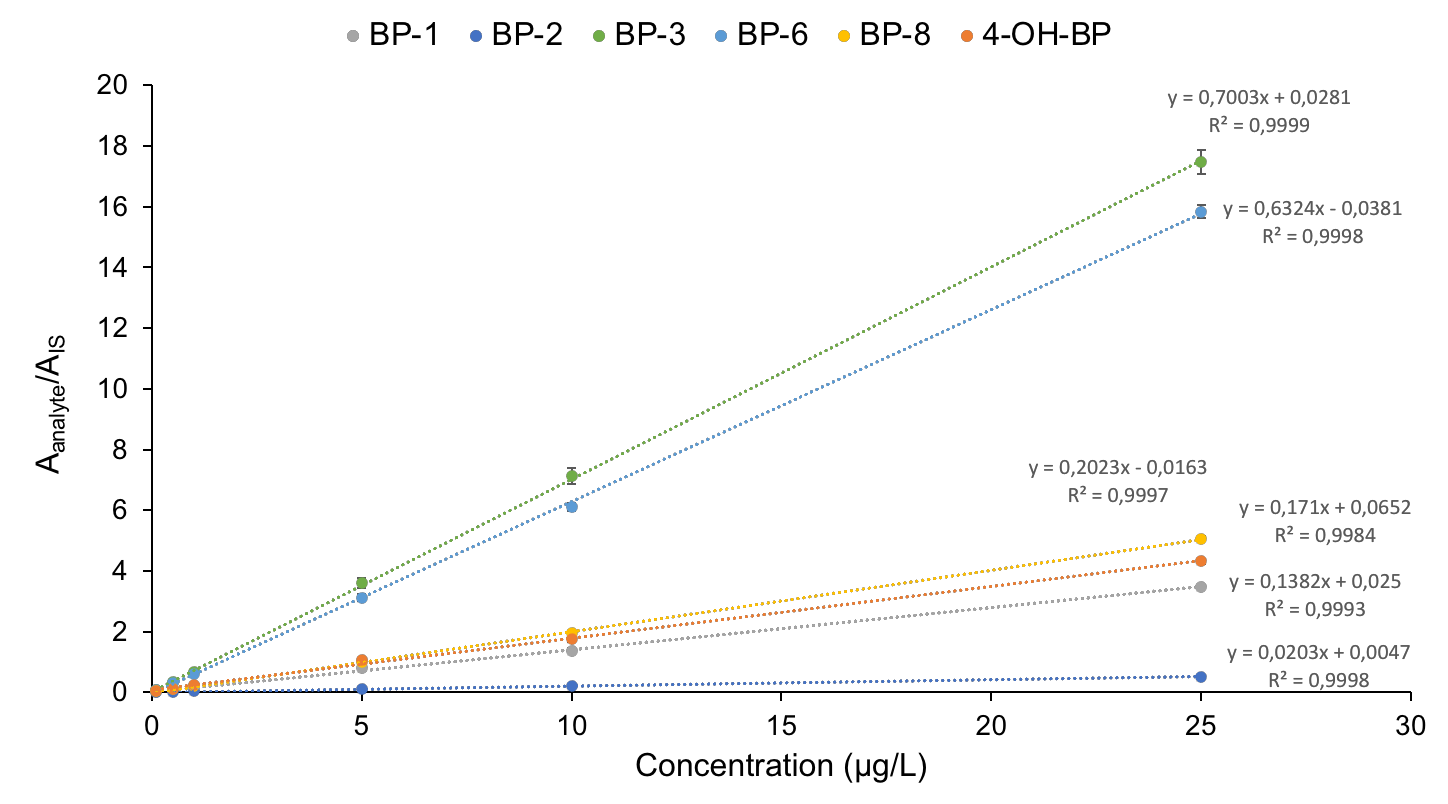


**Fig. S5** Calibration models obtained by plotting the peak area of the analytes divided by that of the internal standard versus the concentration or the target analytes as measured by LC-MS/MS.

**References**

1. Casado-Carmona FA, Alcudia-León M del C, Lucena R, et al (2016) Magnetic nanoparticles coated with ionic liquid for the extraction of endocrine disrupting compounds from waters. Microchem J 128:347–353. https://doi.org/10.1016/J.MICROC.2016.05.011

2. Clarke SJ, Littleford RE, Smith WE, Goodacre R (2005) Rapid monitoring of antibiotics using Raman and surface enhanced Raman spectroscopy. Analyst 130:1019–1026. https://doi.org/10.1039/B502540K

3. Borodko Y, Habas SE, Koebel M, et al (2006) Probing the Interaction of Poly(vinylpyrrolidone) with Platinum Nanocrystals by UV−Raman and FTIR. J Phys Chem B 110:23052–23059. https://doi.org/10.1021/jp063338+

4. Zhang X, Zhou Q, Huang Y, et al (2011) Contrastive Analysis of the Raman Spectra of Polychlorinated Benzene: Hexachlorobenzene and Benzene. Sensors 11:11510–11515. https://doi.org/10.3390/s111211510
